# A genomic‐based vision on the genetic diversity and key performance traits in selectively bred Arctic charr (*Salvelinus alpinus*)

**DOI:** 10.1111/eva.13261

**Published:** 2021-07-02

**Authors:** Christos Palaiokostas, Anam Anjum, Henrik Jeuthe, Khrystyna Kurta, Fernando Lopes Pinto, Dirk Jan de Koning

**Affiliations:** ^1^ Department of Animal Breeding and Genetics Swedish University of Agricultural Sciences Uppsala Sweden; ^2^ Aquaculture Center North Kälarne Sweden

**Keywords:** Arctic charr, ddRAD, genome‐wide association study

## Abstract

Routine implementation of genomic information for guiding selection decisions is not yet common in the majority of aquaculture species. Reduced representation sequencing approaches offer a cost‐effective solution for obtaining genome‐wide information in species with a limited availability of genomic resources. In the current study, we implemented double‐digest restriction site‐associated DNA sequencing (ddRAD‐seq) on an Arctic charr strain with the longest known history of selection (approximately 40 years) aiming to improve selection decisions. In total, 1730 animals reared at four different farms in Sweden and spanning from year classes 2013–2017 were genotyped using ddRAD‐seq. Approximately 5000 single nucleotide polymorphisms (SNPs) were identified, genetic diversity‐related metrics were estimated, and genome‐wide association studies (GWAS) for body length at different time points and age of sexual maturation were conducted. Low genetic differentiation amongst animals from the different farms was observed based on both the results from pairwise *F*_st_ values and principal component analysis (PCA). The existence of associations was investigated between the mean genome‐wide heterozygosity of each full‐sib family (year class 2017) and the corresponding inbreeding coefficient or survival to the eyed stage. A moderate correlation (−0.33) was estimated between the mean observed heterozygosity of each full‐sib family and the corresponding inbreeding coefficient, while no linear association was obtained with the survival to the eyed stage. GWAS did not detect loci with major effect for any of the studied traits. However, genomic regions explaining more than 1% of the additive genetic variance for either studied traits were suggested across 14 different chromosomes. Overall, key insights valuable for future selection decisions of Arctic charr have been obtained, suggesting ddRAD as an attractive genotyping platform for obtaining genome‐wide information in a cost‐effective manner.

## 1 INTRODUCTION

Arctic charr is an attractive candidate for diversifying the Nordic aquaculture industry. An ongoing breeding programme for Arctic charr (*Salvelinus alpinus*) has been operating in Sweden for approximately 40 years resulting in robust improvements of growth rate (Carlberg et al., 2018; Eriksson et al., 2010). The base population of the breeding programme originated from lake Hornavan in Sweden and has been maintained in a closed format with no addition of external germplasm, since the 1980s.

Currently, the Arctic charr breeding programme has been focussed solely on improving growth and delaying sexual maturation. However, reduced reproductive success is currently the main hurdle towards expanding the related farming industry. For instance, survival to the eyed stage has been found to be significantly lower compared to other salmonids (Jeuthe et al., 2016). Available pedigree recordings spanning since the 1980s indicate an average increase of inbreeding since the initiation of the breeding programme equal to approximately 6% (Palaiokostas et al., 2021). Nevertheless, according to pedigree data no clear association has been found so far between the accumulated inbreeding coefficient and survival to critical developmental stages as the eyed stage. Undoubtedly, the preservation of genetic diversity is a critical component for the success of any breeding programme. Interestingly, no genomic information has been used up to date to derive estimates of the breeding nucleus genetic diversity status and potential associations with the low survival to the eyed stage.

Genomic information can be of value for the efficient management and preservation of genetic diversity in farmed animals (Meuwissen et al., 2020; Morales‐González et al., 2020). Moreover, implementation of genomic technologies can allow the discovery of quantitative trait loci controlling traits of interest (Ali et al., 2020; Aslam et al., 2020; Barria et al., 2019; Yoshida & Yáñez, 2021). Overall, only recently high‐throughput sequencing in the form of low coverage genotyping by sequencing has been applied in the case of the Arctic charr breeding programme for managing inbreeding and guiding selection decisions (Palaiokostas et al., 2020). Nevertheless, the above was only applied in a limited number of full‐sib families and therefore not on a fully representative sample of the entire breeding nucleus.

Advancements in the field of genomics over the last decade have boosted aquaculture selective breeding (You et al., 2020). The Atlantic salmon (*Salmo salar*) farming industry, in particular, has benefited tremendously from genomic technologies mainly applied in the form of single nucleotide polymorphisms (SNP) arrays or more recently through whole‐genome sequencing (Houston & Macqueen, 2019). Nevertheless, the aforementioned approaches require considerable financial investment and running costs that are difficult to sustain in the case of aquaculture species where production volume is much lower compared to Atlantic salmon.

Reduced representation sequencing platforms have revolutionized genomic studies of nonmodel organisms offering a cost‐effective alternative for detecting genome‐wide genetic polymorphisms irrespective of the availability of a reference genome (Davey et al., 2011). Following the introduction of restriction site‐associated DNA (RAD) sequencing (Baird et al., 2008), an abundance of reduced representation‐based methodologies is nowadays available using various types‐combinations of restriction enzymes as commonly encountered amongst others in ddRAD‐seq (Peterson et al., 2012), 2b‐RAD (Wang et al., 2012), ezRAD (Toonen et al., 2013), EpiRADseq (Schield et al., 2016), quaddRAD (Franchini et al., 2017) and 2RAD/3RAD (Bayona‐Vásquez et al., 2019). The aforementioned RAD‐derived platforms have proven particularly efficient and flexible as has been demonstrated in a wide range of research studies (Andrews et al., 2016).

As is the case for most emerging aquaculture species, cost‐effective genotyping platforms like RAD appear at the moment as the only realistic solution for advancing the selective breeding programmes to the genomics era. ddRAD‐seq is one of the most popular reduced representation sequencing platforms combining a relatively easy library construction workflow with cost‐efficiency (Peterson et al., 2012). Up to now, ddRAD‐seq has proven to be particularly useful in studying the genetic diversity (Hosoya et al., 2018; Nyinondi et al., 2020; Sherman et al., 2020; Torati et al., 2019) and mapping quantitative trait loci in various fish species (Barría et al., 2018; Brown et al., 2016; Gong et al., 2021; Jiang et al., 2019; Kyriakis et al., 2019; Palaiokostas et al., 2015; Taslima et al., 2020).

The aim of the current study was to gain an in‐depth knowledge regarding the genetic diversity status of the breeding population of Arctic charr and investigate for potential associations between genomic diversity and survival to the eyed stage. Furthermore, we searched for genomic regions associated with important phenotypic traits like growth and sexual maturation. In total, ddRAD‐seq was implemented in 1730 animals from four Arctic charr farms across Sweden. SNPs were detected and used for estimating genetic diversity metrics. Furthermore, mean genetic diversity per full‐sib family of the latest year class (2017) was calculated, and potential associations were investigated with pedigree inbreeding coefficients and survival to the eyed stage. Thereafter, genome‐wide association studies were performed aiming to identify genomic regions associated with growth (in the form of total length) and age of sexual maturation.

## 2 MATERIALS AND METHODS

### 2.1 Ethics statement

All experiments of the current study were performed in accordance with the Swedish legislation described in the Animal Welfare Act 2018:1192 (ethics permit: 5.2.18 – 09859/2019).

### 2.2 Sample collection

Arctic charr samples were collected from four fish farms across Sweden (Arjeplog, Kälarne, Lockne, Timrå). According to information provided by the corresponding fish farm personnel, all tested animals originated from the national breeding programme of Arctic charr which is located at the facilities of Aquaculture Center North (ACN) in Kälarne, Sweden. In addition, no information regarding the year class of the above animals was available. Furthermore, apart from the breeding nucleus site (Kälarne), the other three fish farms use in addition various other strains of Arctic charr, while at the same time no pedigree recordings are being kept. As such, the obtained genotypic data were used to assess whether the sampled animals from Arjeplog (*n* = 48), Lockne (*n* = 16) and Timrå (*n* = 16) originated from the Arctic charr breeding programme (Data S1).

In terms of the sampling conducted at the breeding nucleus site, animals from a range of year classes were used. More specifically, we sampled animals from year classes 2014–2016 (*n* = 70), year class 2013 (*n* = 243) and year class 2017 (*n* = 1337). Animals from year classes 2013 and 2017 were PIT‐tagged and therefore had pedigree data available, while the animals from year classes 2014–2016 did not have accompanying pedigree data. In terms of samples from the 2014–2016 year classes, no information was available regarding their exact year class as those fish were communally reared in a single tank. Regarding the 2017 year class, the sampled animals consisted of 44 full‐sib families with family sizes ranging between 27 and 63 animals. Moreover, available recordings in terms of growth, sexual maturation and survival to the eyed stage were available for the aforementioned full‐sib families (*n* = 1469). In total, fin‐clips for DNA extraction and genotyping were obtained from 1730 animals.

### 2.3 Genomic DNA extraction

A salt‐based precipitation method was applied for extracting genomic DNA. In summary, fin tissue was digested at 55°C (4 hr) using a lysis solution (200 μl SSTNE containing 10% SDS and 100 μg proteinase K). Thereafter, 5 μl RNaseA (2 mg/ml) were added to degrade RNA residues with the samples incubated at 37°C for 60 min. Proteins were precipitated by the addition of 0.7 volume of 5 M NaCl. Genomic DNA was precipitated from the isolated supernatant by addition of 0.7 volume of isopropanol and pelleted by centrifugation. Following overnight incubation in 75% ethanol, the DNA pellet was dissolved in c. 30 μl of 5 mM Tris (pH 8.0). DNA content and quality (260 nm/280 nm and 260 nm/230 nm ratios) was primarily determined by spectrometry (NanoDrop 8000; Thermo Scientific). Thereafter, DNA concentration was measured using the Qubit dsDNA Broad Range Assay Kit. Finally, the samples were diluted to a concentration of 15 ng/ml using 5 mM Tris (pH 8.0) and stored at 4°C before further processing.

### 2.4 ddRAD library preparation and sequencing

In total, 18 ddRAD libraries comprised of 96–98 individuals each were prepared according to Peterson et al. (2012), with minor modifications described in Palaiokostas et al. (2015). In short, each sample (15 ng/μl DNA) was digested at 37°C for 60 minutes with the high‐fidelity enzymes SbfI (recognizing the CCTGCA|GG motif) and NlaIII (recognizing the CATG motif) (New England Biolabs). Individual‐specific combinations of P1 and P2 adapters with a unique 5 or 7 bp barcode (Data S2) were ligated to the digested DNA at 22°C for 120 minutes. Ligation was stopped with the addition of 2.5 volume of PB buffer (Qiagen), and all samples from each library were pooled and purified on a single column (MinElute PCR Purification kit, Qiagen). Thereafter, size selection (400–600 bp) was performed by agarose gel separation followed by gel purification and PCR amplification. A total of 100 μl of each amplified library (13–14 PCR cycles) was purified using an equal volume of AMPure beads (Beckman Coulter). Finally, each library was eluted into 20 μl EB buffer (MinElute Gel Purification Kit, Qiagen) and sequenced in a NovaSeq6000 using seven SP flow cells (150 base paired‐end reads) at the National Genomics Infrastructure centre in Uppsala, Sweden.

### 2.5 Sequence data analysis and SNP genotyping

Reads of low quality (*Q* < 30) and missing the expected restriction sites were discarded. The retained reads were aligned to the *Salvelinus* sp. reference genome assembly [GenBank accession number GCF_002910315] using bowtie2 (Langmead & Salzberg, 2012). Stacks v2.5 (Rochette et al., 2019) was used to extract SNPs using *gstacks* (settings: *var‐alpha 0.01*; *gt‐alpha 0.01*). In the case where a single ddRAD locus had multiple SNPs, only the first encountered SNP was used for downstream analysis (*‐‐write‐single‐snp*). SNPs with minor allele frequency (MAF) <0.05, found in less than 30% of the animals in each population and with mean heterozygosity >0.7 across the tested samples, were discarded using the *populations* module of Stacks v2.5. Finally, SNPs deviating from Hardy–Weinberg equilibrium (HWE) were discarded using the preGSf90 v1.21 software from the BLUPF90 suite (Misztal et al., 2018). More specifically, SNPs, where the number of observed heterozygotes deviated above a threshold of 0.15 from the number of expected heterozygotes under HWE, were discarded (Wiggans et al., 2009).

### 2.6 Genetic diversity—population differentiation

Genetic variation metrics like mean observed (*H*_o_) and expected (*H*_e_) heterozygosity per population were estimated using the Stacks software v2.5 (Rochette et al., 2019). Principal component analysis (PCA) was conducted using the R package adegenet v2.1.1 (Jombart, 2008) for gaining insights of the underlying genetic structure amongst animals from the different fish farms. Pairwise fixation index (*F*_st_) values between all tested populations were estimated using the R package StAMPP v.1.6.1 (Pembleton et al., 2013). Due to the uneven sample size of each year class and in order to avoid overwhelming, our population differentiation results by the large number of samples from the 2017 year class the *F*_st_ estimates and the PCA did not include those animals.

### 2.7 Genetic diversity per full‐sib family (2017 year class) and associations with inbreeding and eyed stage survival

The range of *H*_o_ for each full‐sib family of the 2017 year class was estimated using the R v.4.0.0 programming language (R Core Team, 2019). In addition, inbreeding coefficients for the aforementioned animals were estimated using a recursive algorithm assuming nonzero inbreeding of unknown parents (Aguilar & Misztal, 2008) as implemented in the INBUPGF90 v1.43 software from the BLUPF90 suite (Misztal et al., 2018). The Pearson correlation coefficient was used to investigate for linear relationships between the genetic diversity status of each full‐sib family and the corresponding inbreeding coefficient. Finally, correlations were estimated between the percentage of embryo survival to the eyed stage for each full‐sib family and the corresponding genetic diversity (mean *H*_o_) or inbreeding coefficient.

### 2.8 Phenotypic recordings of full‐sibs from the 2017 year class

Growth measurements, including body weight and total length, were taken on three occasions from each individual fish during 2018 and 2019. In particular, measurements were taken in January 2018, September 2018 and September 2019 (Data S3). On all recordings, body weight and standard length were recorded to the closest gram and millimetre, respectively. From the available growth‐related traits in the current study, we focussed only on total length due to the fact that it is highly correlated with body weight, while at the same time reduces the possibility of obtaining animals with rounder body shapes (Schaeffer et al., 2018). Pearson's correlation coefficients were estimated amongst the different time points of each recorded growth trait using R v.4.0.0 (R Core Team, 2019). Moreover, during the reproductive season of 2019 (October–November) phenotypic sex was recorded from the 2^+^ year of age animals assigning them to males, females or immature‐sterile. In total, recordings from 1469 animals were obtained.

### 2.9 Genetic parameter estimation—Genome‐wide association analysis in 2017 year class

Heritability estimates related to growth have been recently estimated in our previous study (Palaiokostas et al., 2021). More specifically, *h* ^2^ ranged between 0.27 and 0.33 for the different time point measurements in the case of body length. In the current study, variance components were estimated for sexual maturation using THRGIBBS1F90 (Misztal et al., 2018) using the following animal model:

(1) y=Xb+Zu+e

where **b** is the vector of the fixed effects (intercept, total length, tank); **X** is the incidence matrix relating phenotypes with the fixed effects; **Z** is the incidence matrix relating phenotypes with the random animal effects; **u** is the vector of random animal effects ~N(0, **G** $\sigma_{g}^{2}$) where **G** is the genomic relationship matrix (VanRaden, 2008), $\sigma_{g}^{2}$ is the additive genetic variance; **e** the vector of residuals ~N(0, **I** $\sigma_{e}^{2}$) and $\sigma_{e}^{2}$ is the residual variance.

The parameters of this model were estimated through Markov chain Monte Carlo (MCMC) using Gibbs sampling (11 M iterations; burn‐in: 1 M; thin: 1000). Heritability was estimated using the following formula:

$$h^{2}=\frac{\sigma_{g}^{2}}{\sigma_{g}^{2}+\sigma_{e}^{2}}$$

The probit link function was used to connect the observed binary phenotype (0 = Sexually mature, 1 = Nonsexually mature) with the underlying liability scale. Residual variance on the underlying scale is not identifiable in threshold models (Goldstein et al., 2002; Nakagawa & Schielzeth, 2013) and was therefore fixed to 1.

Thereafter, a genome‐wide scan was conducted in order to look for genomic regions associated with either the growth trait (total length) or maturation status at 2^+^ years of age. More specifically, a weighted single‐step genomic best linear unbiased prediction (WssGBLUP) analysis was performed (Wang et al., 2012) using preGSf90 v1.21 and postGSf90 v1.70 from the BLUPF90 software suite. Moreover, in the case of length, the breeding values were estimated using BLUPF90 v1.69, while in the case of sexual maturation, the breeding values were estimated using the THRGIBBS1F90 v2.116. A genomic relationship matrix was estimated following VanRaden (2008) as:

$$G=\frac{Z\;DZ^{\prime }}{2\sum p_{i}\left(1‐p_{i}\right)}$$

where **Z** is a matrix of centred genotypes, **D** is a weight matrix for all SNPs and *p_i_* the corresponding MAF for each SNP. Thereafter, SNP weights were calculated using the nonlinearA method (VanRaden, 2008). The steps for performing WssGBLUP included the following (Zhang et al., 2016):

1. Initialization of **D** = **I** and *t* = 1, where **I** the identity matrix and *t* is the iteration number.
2. Calculation of **G**.
3. Estimation of genomic breeding values (GEBVs).
4. Estimation of SNP effects from GEBVs: $\hat{\alpha }=qDZ^{\prime }G*\hat{u}$, where $\hat{\alpha }$ the vector of SNP effects and $\hat{u}$ the vector of GEBVs.
5. Calculation of individual SNP weights: $d_{\mathrm{ii}}^{\left(t+1\right)}=1.125^{\frac{\left|{\hat{a}}_{i}\right|}{sd(\hat{a})}‐2}$, where ${\hat{a}}_{i}$ the estimated SNP effect using the nonlinearA approach (VanRaden, 2008).
6. Normalization of SNP weights so the total genetic variance remains constant.
7. Estimation of $G^{t+1}=\frac{Z\;D^{t+1}Z^{\prime }}{2\sum p_{i}\left(1‐p_{i}\right)}$
8. Iteration from step (3) until convergence (10^−14^).

Convergence of SNP weights was tested using the following criterion

$$C=\frac{\sum_{i}{\left(w_{i}‐w_{i‐1}\right)}^{2}}{\sum_{i}w_{i}^{2}}$$

where *w_i_* refers to the estimated SNP weight amongst iterations.

Finally, the percentage of explained additive genetic variance was estimated by nonoverlapping windows of 10 adjacent SNPs as follows:

$$\frac{\text{var}\left(\alpha_{i}\right)}{\sigma_{g}^{2}}\times 100\%=\frac{\text{var}\left(\sum_{i}^{i=10}z_{i}{\hat{\alpha }}_{i}\right)}{\sigma_{g}^{2}}\times 100\%$$

where var(*α_i_*) the additive genetic variance of the tested window of adjacent SNPs, $\sigma_{g}^{2}$ the total additive genetic variance, *z_i_* the genotype of each of the 10 individual SNPs located in the tested window and *α_i_* the corresponding SNP effect.

## 3 RESULTS

### 3.1 Dataset filtering

In total, 1730 Arctic charr individuals were genotyped using ddRAD. Approximately 5.4 billion 150 bp long paired‐end reads were obtained. Overall, approximately 74% of the obtained reads (~4 billion reads) passed all quality control filtering steps and were retained. Sequencing of 12 samples failed with 1718 animals being retained. The number of reads passing quality filters per sample ranged between ~200 thousand and 11 million with a mean and standard deviation of 2.4 million and 1.2 million reads, respectively (Figure S1).

**FIGURE 1:**
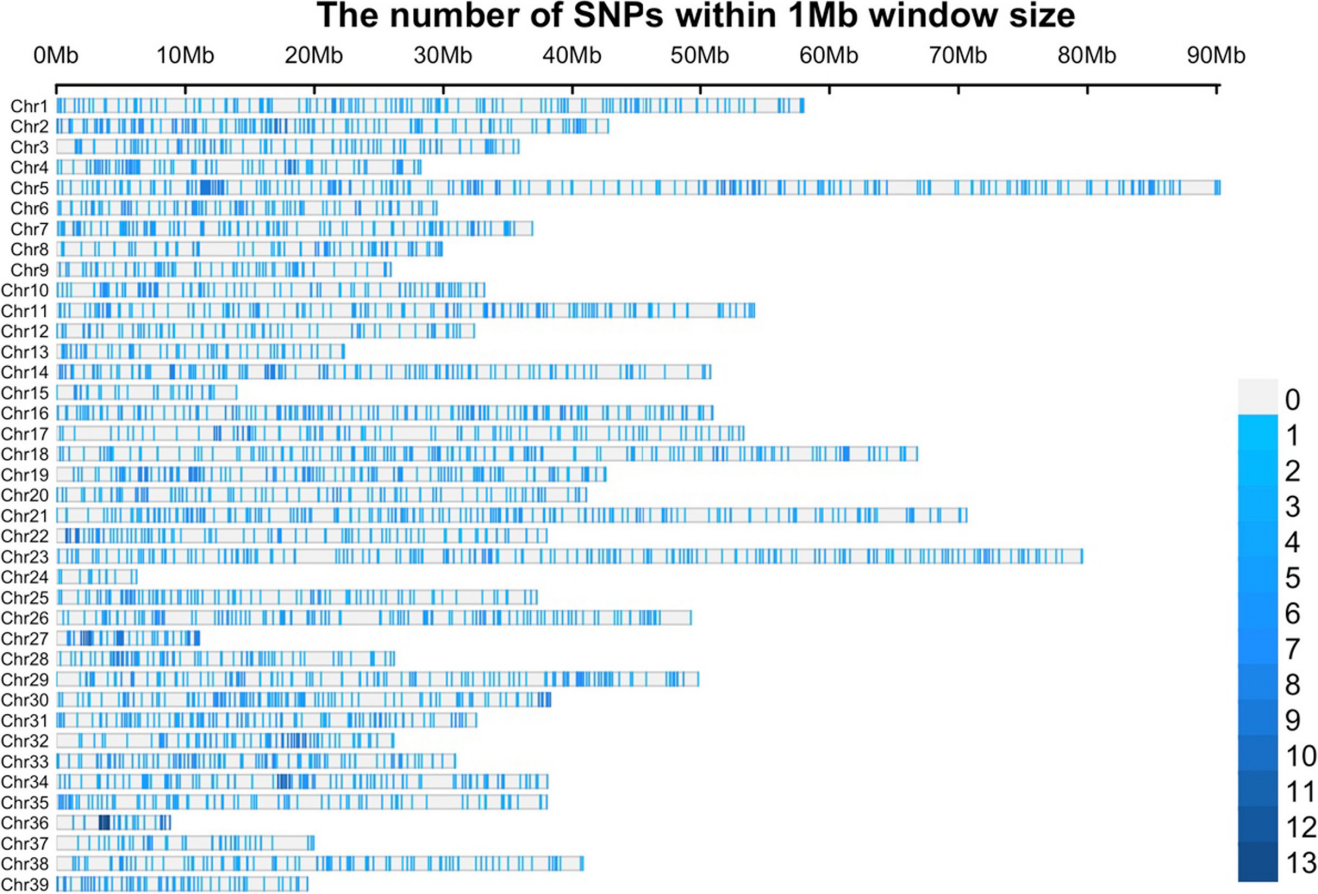
Location and density of the detected single nucleotide polymorphisms (SNPs) in the genome of *Salvelinus* sp

Thereafter, approximately 28 thousand putative loci were identified with a mean coverage per sample being equal to 47× (SD 19×). In total, 5072 SNPs fulfilled the QC criteria (MAF, missing data, HWE) and were kept for downstream analysis. From the identified SNPs, 4009 were located in the 39 assembled chromosomes of the *Salvelinus* sp. reference genome, while the remaining 1063 were found in unplaced contigs (Figure 1). Finally, 48 animals were removed due to low calling rate (<70%) resulting in a final dataset consisting of 1670 animals being genotyped for 5072 SNPs.

### 3.2 Genetic diversity between animals from different fish farms

**FIGURE 2:**
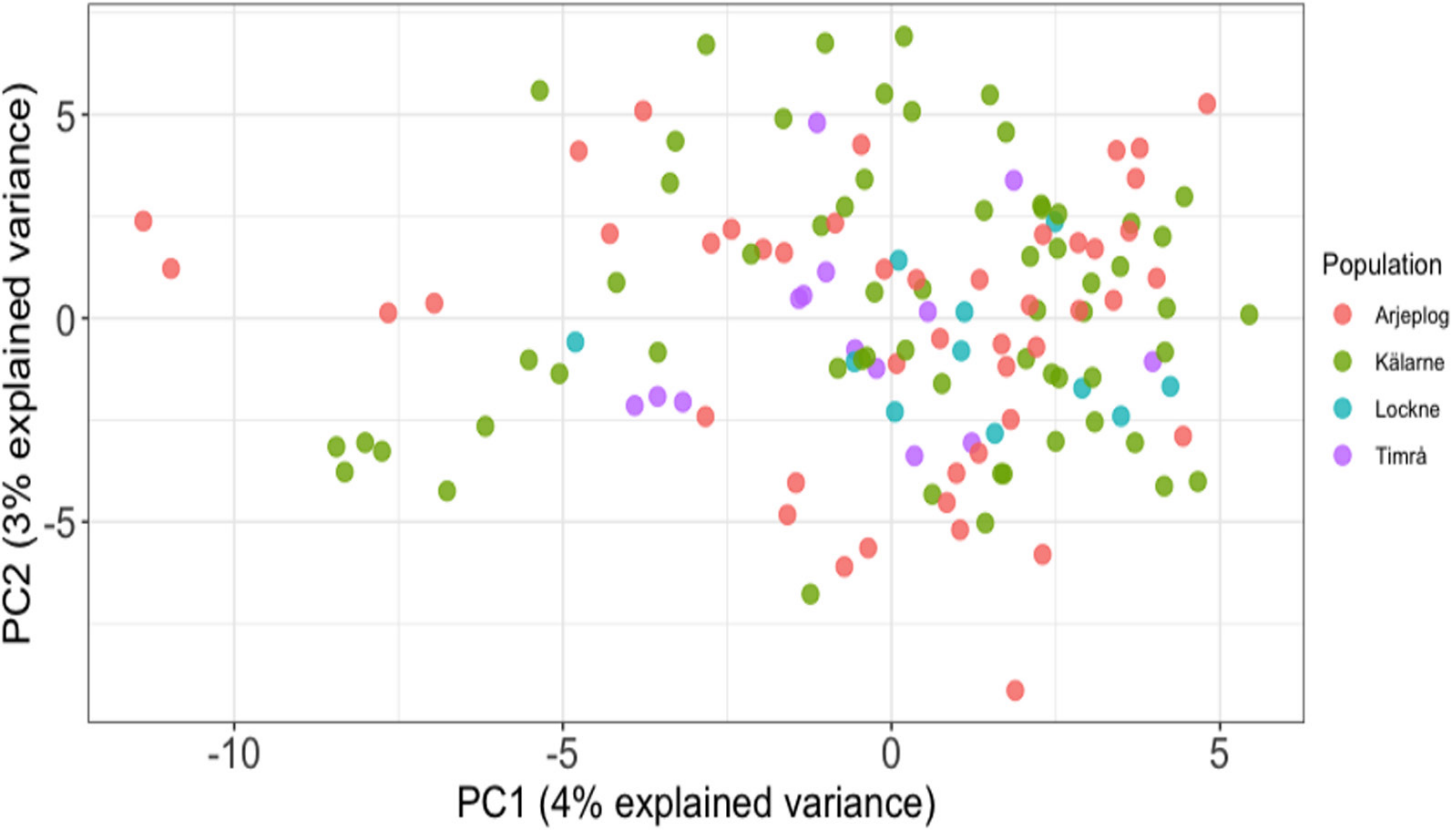
Principal component analysis on animals sampled across four different sites

**FIGURE 3:**
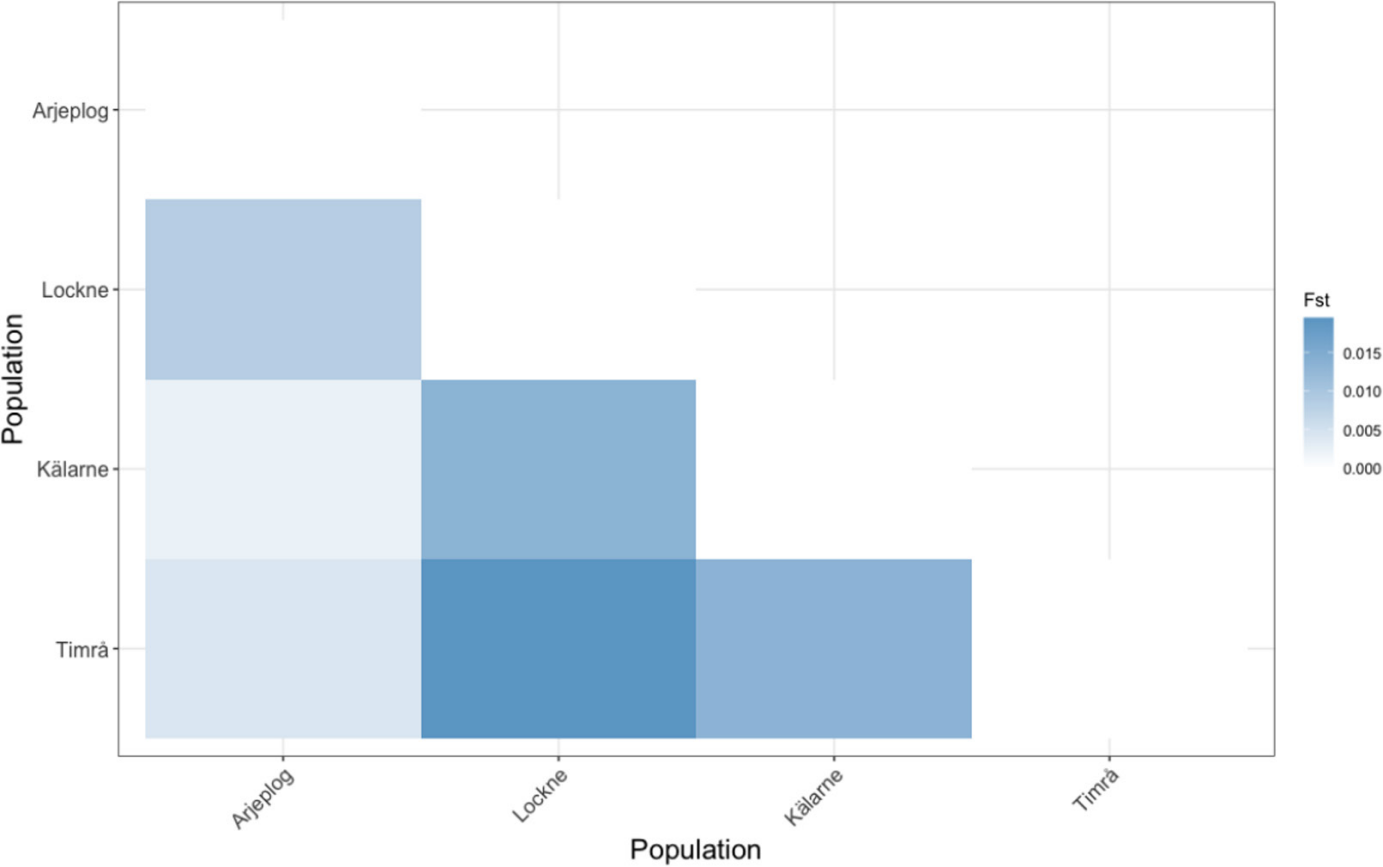
Pairwise *F*_st_ values amongst the sampled populations

The number of sampled animals from the different farms varied substantially (Table 1) with Lockne being the population with the lowest sample size (*n* = 12), while on the other hand, Kälarne was the population with the highest number of animals (*n* = 277). The mean observed heterozygosity (*H*_o_) for the genotyped Arctic charr samples was 0.34, while the mean expected heterozygosity (*H*_e_) was 0.37. The Timrå population had the highest levels of *H*_o_ (0.39), while the other three populations had a *H*_o_ of 0.33–0.37 (Table 1). PCA was used to visualize the relationships within and between the sampled populations. The first and second principal components accounted for 4% and 3% of the total variation, respectively (Figure 2). Pairwise *F*_st_ indices amongst the tested populations were estimated to infer the genetic distance amongst the sampled populations. Low levels of genetic differentiation amongst the tested populations were obtained with *F*_st_ values ranging between 0.002 and 0.02 (Figure 3). Based on the *F*_st_ estimates, the highest genetic distance was observed between the populations from Timrå and Lockne and the lowest between Kälarne and Arjeplog.

**TABLE 1:** Origin of Arctic charr passing quality control and estimates of their genetic diversity

Population	No. animals	*H*_o_	*H*_e_
Arjeplog	46	0.33	0.34
Lockne	12	0.32	0.33
Timrå	15	0.39	0.33
Kälarne YC2013	223	0.34	0.35
Kälarne YC2014‐2016	54	0.33	0.34
Kälarne YC2017	1320	0.37	0.35

### 3.3 Genetic diversity per full‐sib family (2017 year class)—association with eyed stage survival

**FIGURE 4:**
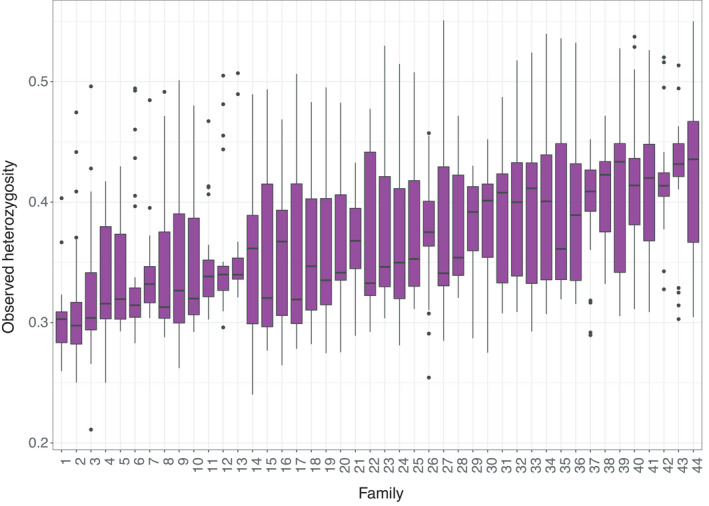
Observed heterozygosity values for each full‐sib family of the 2017 year class

**FIGURE 5:**
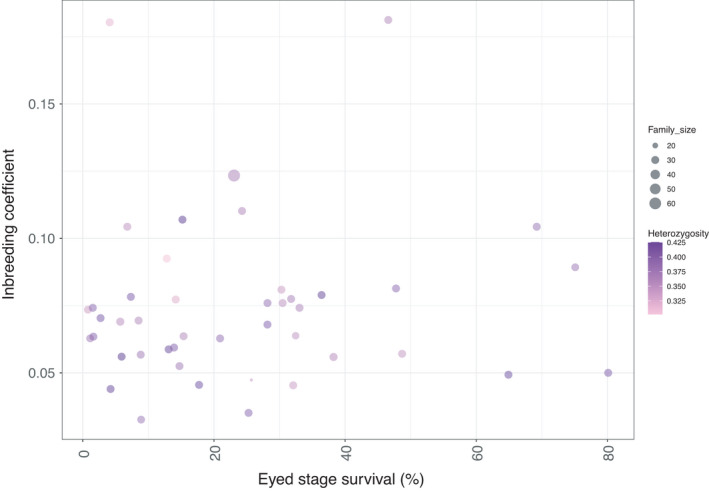
Scatter plot of mean observed heterozygosity and inbreeding coefficient on eyed staged survival

Genetic diversity within each full‐sib family from the 2017 year class was estimated in terms of *H*_o_. Furthermore, the inbreeding coefficient of each full‐sib family was inferred from the available pedigree recordings. The mean *H*_o_ metric amongst the 44 full‐sib families ranged between 0.30 and 0.42 (Figure 4) with a mean value of 0.37, while the corresponding inbreeding coefficient ranged between 0.03 and 0.18 with a mean value of 0.07. A moderate correlation equal to −0.33 was obtained between the mean *H*_o_ and the inbreeding coefficient of each family. On the other hand, a correlation close to zero was obtained between either *H*_o_ or the inbreeding coefficient and the eyed stage embryo survival (Figure 5).

### 3.4 Exploratory analysis of key production traits in 2017 year class Arctic charr

**FIGURE 6:**
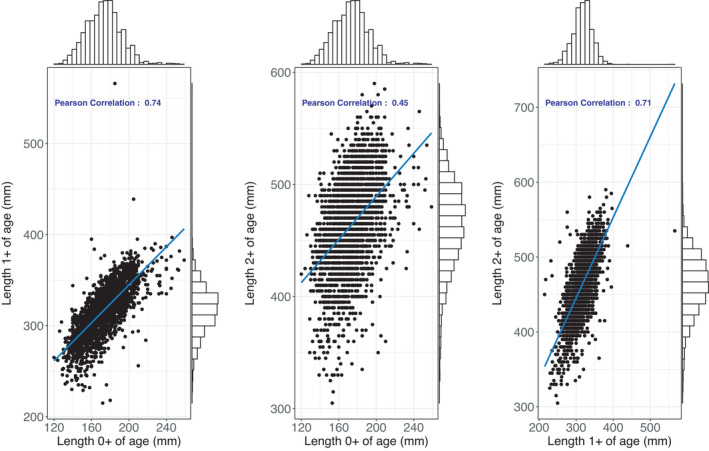
Phenotypic distribution and correlations between total body length recorded at three different time points

**FIGURE 7:**
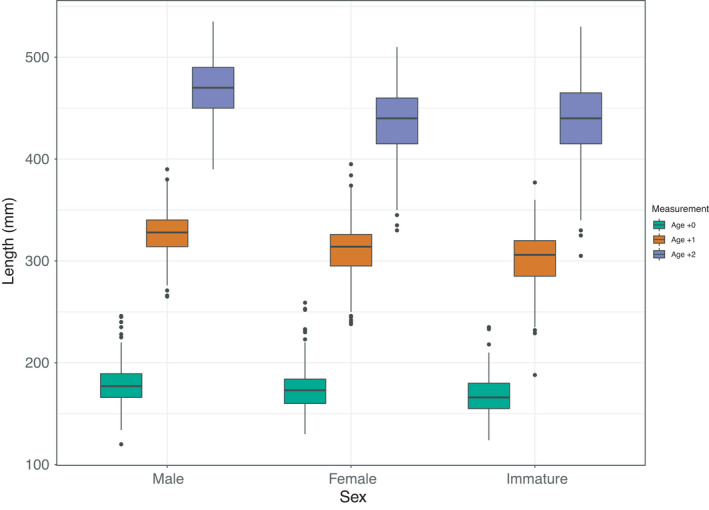
Distribution of total body length across phenotypic sex on three time points

Recordings regarding growth (total length in mm), phenotypic sex and sexual maturation were available from 1469 Arctic charr individuals. Total length in the three different time point recordings ranged between 120 and 259, 215 and 395 and 305 and 560 mm, respectively. The correlation between the three recordings ranged between 0.45 and 0.74 (Figure 6). Regarding the phenotypic sex of the animals from the 2017 year class, 608 and 516 were classified as females and males, respectively. On the other hand, 345 were classified as immature at 2^+^ year of age. A consistent sexual dimorphism in favour of the males was obtained across all three time point measurements ranging between 3% and 8%. In the first two measurements, the sexually immature animals had the smallest size on average as opposed to the ones being classified as either males or females. During the final time point measurement, the mean size of the sexually immature animals was close to the size of the females (Figure 7).

### 3.5 Heritability for age of sex maturation—Genome‐wide association analysis

**FIGURE 8:**
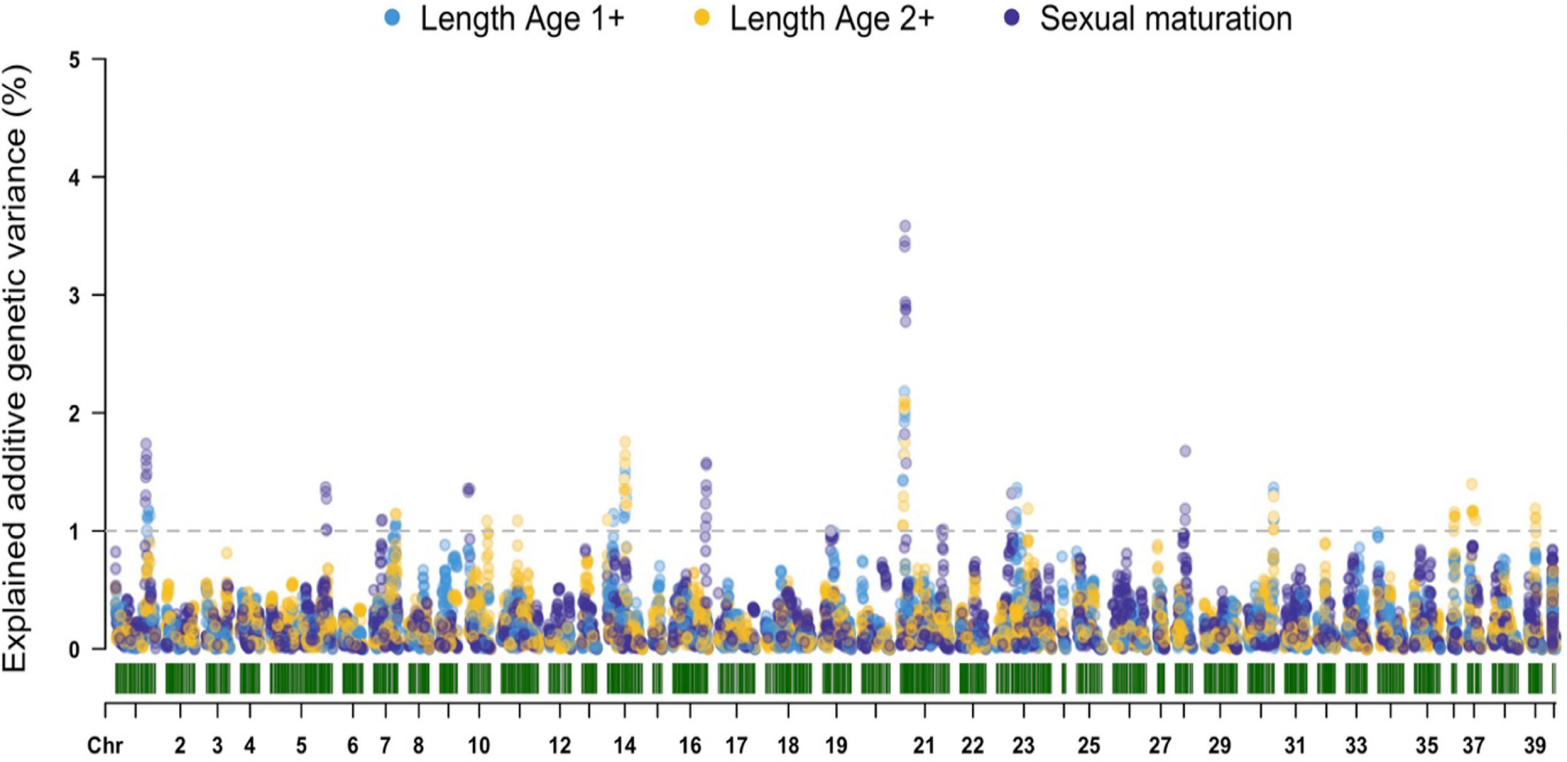
Genome‐wide association studies using WssGBLUP for total body length and sexual maturation

A moderate heritability for age of sex maturation equalled to 0.28 (HPD 95% 0.16–0.41) on the underlying scale was obtained. Out of 1469 animals with available phenotypic recordings (total length, sexual maturation), 1320 had corresponding genotypic data derived from ddRAD. As such, WssGBLUP was preferred in order to use all the available information. Due to the low phenotypic correlation between length at the first and last time points, we conducted WssGBLUP only on the last two time points due to their higher relevance with actual harvest sizes. The convergence of the WssGBLUP algorithm was achieved after two iterations using the nonlinearA approach to estimate SNP effects. Genomic regions explaining more than 1% of the additive genetic variance for the tested traits were detected in 14 different chromosomes out of which three (chromosomes 7, 21 and 23) included all three tested traits. The region with the highest percentage of explained additive genetic variance was found in chromosome 21, where all three tested traits explained more than 3% of the additive genetic variance (Figure 8).

## 4 DISCUSSION

Reduced representation sequencing in the form of ddRAD was applied in the current study, aiming to provide insights related to the genetic diversity status and detect genomic regions associated with important phenotypic traits in selectively bred Arctic charr. In contrast to other salmonids like Atlantic salmon (*Salmo salar*) and rainbow trout (*Oncorhynchus mykiss*), genome‐wide related resources for Arctic charr are starting to appear only in the last couple of years (Nugent et al., 2019). Furthermore, in comparison to Atlantic salmon and rainbow trout, a wide range of Arctic charr strains are being farmed worldwide, including both anadromous and landlocked populations. As such, especially in the Arctic charr case reduced representation sequencing platforms like ddRAD are particularly useful as in addition to the significantly lower economic costs for initial development compared to SNP arrays, the RAD‐type approaches do not tend to suffer from SNP ascertainment bias (Robledo et al., 2017).

In terms of prior genomic studies on the Swedish selectively bred Arctic charr strain, limited research has been conducted so far and as such available genomic resources are scarce. It has to be stressed that the aforementioned strain has the longest selection history amongst all known selected populations of Arctic charr worldwide. Therefore, most interesting opportunities exist for studying critical evolutionary mechanisms like selection signatures along the genome as in other fish species with long history of selection (Gutierrez et al., 2016; López et al., 2014). Using high‐throughput sequencing, we recently applied low coverage genotyping by sequencing in animals from the Swedish Arctic charr breeding programme (Palaiokostas et al., 2020). Nevertheless, the aforementioned study included only a small subset of full‐sib families (*n* = 13) limiting our ability to derive conclusions regarding amongst others the genetic diversity status of the breeding nucleus. Furthermore, all the above animals were sampled only from the breeding nucleus. The identified SNPs (~5000) of our study that were detected in a large number of animals spanning through different year classes (2013–2017) provide a valuable resource for all sort of future genomic studies on the selected strain of Arctic charr.

### 4.1 Genetic differentiation between Arctic charr sampled from different fish farms

Over the years, germplasm from the Arctic charr breeding programme has been disseminated to farms across the country. Nevertheless, since other strains of Arctic charr are also reared in Sweden, it is not always clear whether admixture has taken place with the selectively bred animals. Since tracking individual fish through PIT‐tagging is not usually performed in commercial farms, the usage of genetic markers appears to be the only alternative for deciphering the genetic diversity status of the reared stocks. Samples from three other commercial Arctic charr farms were collected in addition to the sampling that was performed in the breeding nucleus site. Even though, in our analysis we were able to sample only a small number of animals from two of the four sampled farms (*n* = 12–15) both PCA and the derived *F*_st_ values indicated a minor genetic differentiation amongst the sampled populations confirming our prior information that all sampled animals originated from the Arctic charr breeding programme. Even though no substitute exists for the ideal scenario where the genetic differentiation analysis is based on large sample sizes for all the tested populations, the *F*_st_ index is relatively resilient to the effects of sample size (Willing et al., 2012). Furthermore, with the exception of the pairwise comparison between samples from Lockne and Timrå (populations with lowest number of sampled individuals) the remaining *F*_st_ values were below 0.01. To bring the above values into context, a recent large‐scale study (Ferchaud et al., 2020) conducted on 1416 Brook charr (*Salvelinus fontinalis*) individuals reported *F*_st_ values amongst the studied populations that ranged between 0.03 and 0.53 (median = 0.28).

### 4.2 Genetic diversity status of the breeding nucleus

Since preservation of genetic variation is critical for the viability and long‐term success of any breeding programme, in the current study, we placed particular emphasis on the genetic diversity status of the breeding nucleus focussing primarily on the latest available year class from 2017 (*n* = 1337; 44 full‐sib families). Moreover, using pedigree data we were able to investigate for potential associations between heterozygosity estimates and the corresponding pedigree derived inbreeding coefficients. Overall, the range of observed heterozygosity amongst the 44 full‐sib families of the 2017 year class was in accordance with previous reduced representation sequencing studies on fish (Saenz‐Agudelo et al., 2015) and with our previous study on Arctic charr were a different genotyping by sequencing library preparation approach was followed (Palaiokostas et al., 2020). Notably, a slight heterozygote excess (*H*_o_ > *H*_e_) was observed in the case of the 2017 year class which at first sight might appear counterintuitive for the case of a closed breeding nucleus. However, a similar phenomenon with similar heterozygosity estimates was observed in selectively bred rainbow trout with a possible explanation being a recent bottleneck (<100 generations ago) reflecting the relatively recent domestication and selection process (D'Ambrosio et al., 2019). In our case, the slight heterozygote excess was not uniformly observed across all the tested year classes though which could be due to the unequal sample size as the 2017 year class included more than 70% of the animals that were genotyped in our study.

Despite the fact that it could be argued that potential attempts to predict fitness traits using genetic diversity indexes like observed or expected heterozygosity would not be informative, genome‐wide heterozygosity has been hypothesized to be associated with fitness‐related traits in various organisms including fish (Morris et al., 2019). In the current study, no associations were observed between the estimated genome‐wide heterozygosity and corresponding phenotypic traits of interest like survival of Arctic charr embryos to the eyed stage. However, a moderate negative correlation coefficient was estimated (−0.33) between observed heterozygosity and the corresponding inbreeding coefficient. Since the level of inbreeding is a pivotal parameter in selective breeding, it would be particularly interesting to monitor the subsequent generation(s) of selectively bred Arctic charr in terms of genetic diversity and related inbreeding levels.

### 4.3 Genome‐wide association studies on commercially important traits

In the current study, we looked for genomic regions associated with economically important traits such as growth and sexual maturation. It should be noted that no premature sexual maturation recordings were used. Premature males are currently found at very low frequency in the Arctic charr breeding programme since they are negatively correlated with growth (Nilsson et al., 2010). Interestingly, sexual growth dimorphism was observed in Arctic charr in favour of males (Palaiokostas et al., 2021) showing a similar pattern as in the Norwegian Atlantic salmon (Thorland et al., 2020). Notably, sexual growth dimorphism in our study was observed even at the early life stages with the animals classified as sexually immature (when more than two years of age) demonstrating the lowest growth potential.

No locus of major effect was detected for the tested traits (total length at two different time points and sexual maturation at 2^+^ years of age). Even though the above is to be expected for growth‐related traits, major loci associated with sexual maturation have been previously documented. More specifically, the *vgll3* gene has been found to control the age of sexual maturation in anadromous Atlantic salmon males (Ayllon et al., 2015; Barson et al., 2015). However, in the current study, we used sexual maturation recordings related to both females and males. In addition, since the genotyping density was not particularly high (~5000 SNPs) there is the possibility that loci of effect remained undetected. Overall though, a high number of putative important genomic regions with effect were detected accounting for ~15%–20% of the additive genetic variance which could be of value in guiding future selection decisions in the Arctic charr breeding programme. Additionally, a moderate heritability estimate was obtained in the case of sexual maturation indicating that this trait is amenable to selection.

As the number of available phenotypic recordings for the 2017 year class was higher (*n* = 1469) than the corresponding genotypic information (*n* = 1320), a WssGBLUP approach (Zhang et al., 2016) was undertaken. WssGBLUP has been routinely applied in genome‐wide association studies in various fish breeding studies for detecting genomic regions associated with traits of interest (Ali et al., 2020; Barria et al., 2019; Garcia et al., 2018; Salem et al., 2018; Vallejo et al., 2019; Yoshida et al., 2019). Approaches like WssGBLUP are particularly appealing in breeding studies where only a subset of the available pedigree data is genotyped, as they allow the usage of all the available information including pedigree relationships, phenotypic and genotypic recordings (Misztal et al., 2020). However, a potential issue with WssGBLUP lies on the nontrivial task of deciding upon the optimal number of iterations and weights for each SNPs. The approaches that were initially suggested (e.g., SNP variance based on squared SNP effects) required to a priory decide on the number of iterations since otherwise excessive values of the explained variance would be obtained. Nevertheless, using the nonlinearA method (VanRaden, 2008) we were able to avoid the aforementioned issues (Lourenco et al., 2020). In the current study, convergence was obtained within two iterations with the obtained estimates being stable when a higher number of iterations was tested (3–5).

## 5 CONCLUSIONS

Genome‐wide information was obtained in the current study aiming to investigate the genetic diversity status of selectively bred Arctic charr from different year classes. Even though no signs of inbreeding depression were observed, continuously monitoring the genetic diversity of the selected population using genomic information is expected to benefit the breeding programme. A genome‐wide association approach with WssGBLUP did not unveil loci with major effect for the tested traits. Nevertheless, genomic regions with potential interest for the studied traits were detected. Overall, the routine implementation of reduced representation genotyping by sequencing platforms like ddRAD can benefit the selection decisions by offering a high resolution in terms of individual genetic diversity levels of each breeding candidate and provide insights about the underlying genetic architecture for traits of interest.

## CONFLICT OF INTEREST

The authors declare no conflict of interest.

## Supporting information

Figure S1

Data S1

Data S2

Data S3

## Data Availability

Sequence information of the utilized samples was deposited in the National Centre for Biotechnology Information (NCBI) repository in bam format under project ID PRJNA695412.
