# Supplementary figures and images for: A genomic‐based vision on the genetic diversity and key performance traits in selectively bred Arctic charr (Salvelinus alpinus)
Source: Evol Appl. 2021 Jul 2;15(4):565–77. doi: 10.1111/eva.13261 (PMC9046918; doi:10.1111/eva.13261)

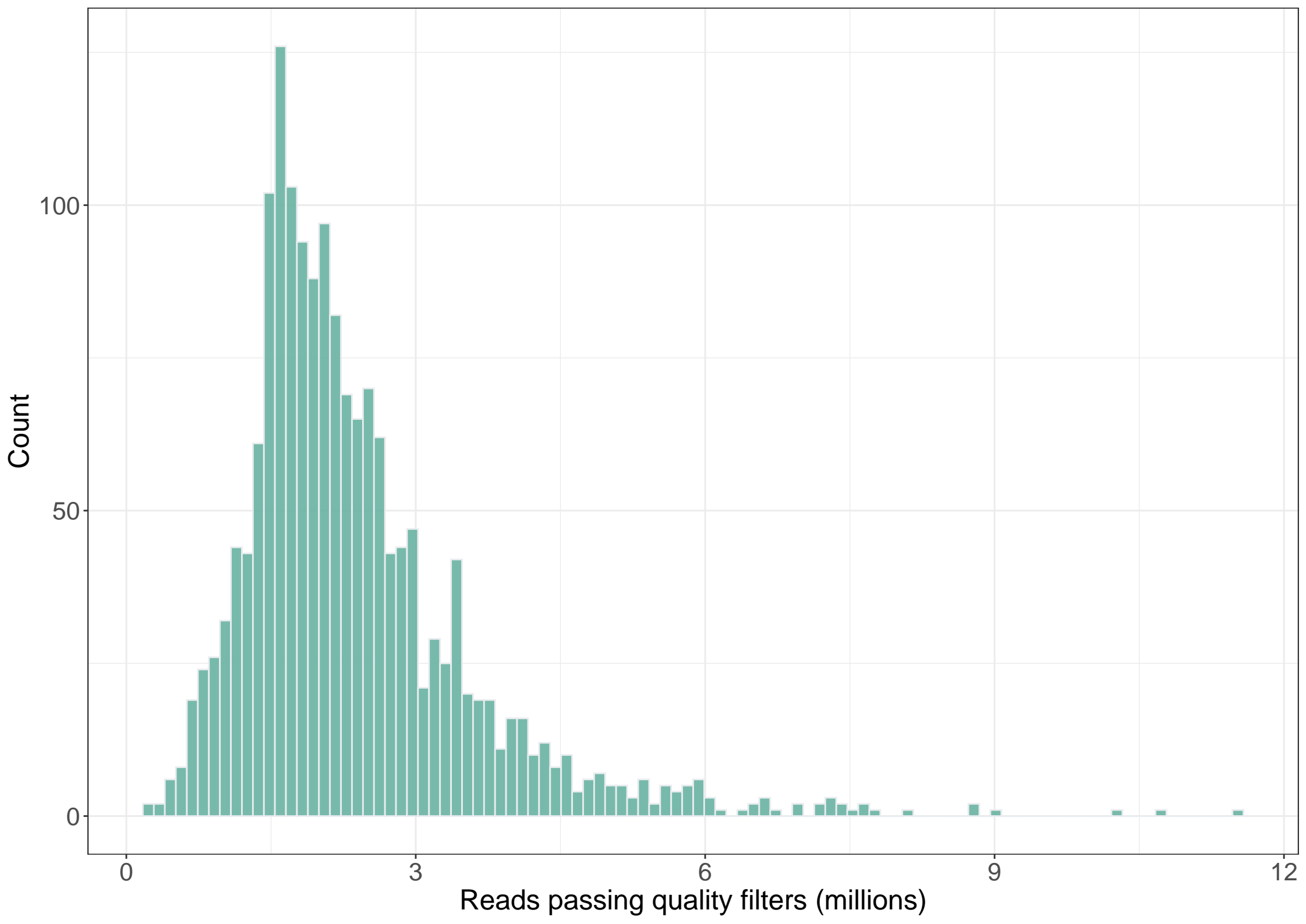

Supplement: Supplementary file 1 — Figure S1 [file EVA-15-565-s001.pdf]
